# Exploring the metabolic signature of intermittent explosive disorder: Preliminary evidence and potential mechanisms for altered bilirubin metabolism

**DOI:** 10.1016/j.cpnec.2025.100294

**Published:** 2025-04-22

**Authors:** Jeffrey B. Schwimmer, Murray B. Stein, Emil F. Coccaro, Alejandro D. Meruelo

**Affiliations:** a Division of Gastroenterology, Hepatology, and Nutrition, Department of Pediatrics, University of California San Diego School of Medicine, La Jolla, CA, USA; b Department of Gastroenterology, Rady Children's Hospital, San Diego, CA, USA; c Department of Psychiatry, University of California San Diego, 9500 Gilman Drive, La Jolla, CA, 92093, USA; d Psychiatry Service, Veterans Affairs San Diego Healthcare System, San Diego, CA, USA; e Clinical Neuroscience and Psychotherapeutics Research Unit, Department of Psychiatry and Behavioral Health, The Ohio State University Wexner Medical Center, Columbus, OH, USA; f University of California San Diego, 9500 Gilman Drive, La Jolla, CA, 92093, USA

**Keywords:** Intermittent explosive disorder, Bilirubin metabolism, Hepatobiliary function, Oxidative stress, Systemic biomarkers, Metabolic vulnerability

## Abstract

Intermittent Explosive Disorder (IED) is characterized by impulsive aggression and emotional dysregulation, yet its systemic biological underpinnings remain poorly understood. This study examined bilirubin metabolism and systemic biomarkers as indicators of metabolic vulnerability in individuals with IED. Laboratory data for total and indirect bilirubin and white blood cell (WBC) count were analyzed in individuals with IED and a demographically and clinically matched general population (GP) control group. A 10:1 nearest-neighbor propensity score matching procedure was used to balance covariates including age, sex, race, ethnicity, body mass index (BMI), and alcohol and tobacco use. Participants with hepatobiliary or inflammatory conditions were excluded to reduce heterogeneity and confounding. Group comparisons used unique individuals with biomarker values averaged across timepoints.

Individuals with IED showed lower total and indirect bilirubin levels compared to matched controls, with a moderate effect size for indirect bilirubin (d = −0.37) and a small effect for total bilirubin (d = −0.10). WBC differences were minimal (d = −0.12). Linear mixed-effects models incorporating repeated measures yielded consistent results, though none of the group differences reached statistical significance, likely due to limited sample size in the IED group. Sensitivity analyses suggested bilirubin findings were more robust to unmeasured confounding than WBC.

These results highlight a potential hepatobiliary or metabolic signature in IED, rather than a primary inflammatory process. Given the preliminary nature of the findings, absence of cytokine data, and limited statistical power, results should be interpreted cautiously and warrant replication in larger samples with broader inflammatory and lifestyle profiling.

## Highlights

• Individuals with IED showed lower total and indirect bilirubin than matched controls.
• Linear mixed-effects models supported modest bilirubin reductions in IED.
• Preliminary findings highlight bilirubin as a potential metabolic biomarker in IED.

## Graphical abstract

**Figure:**
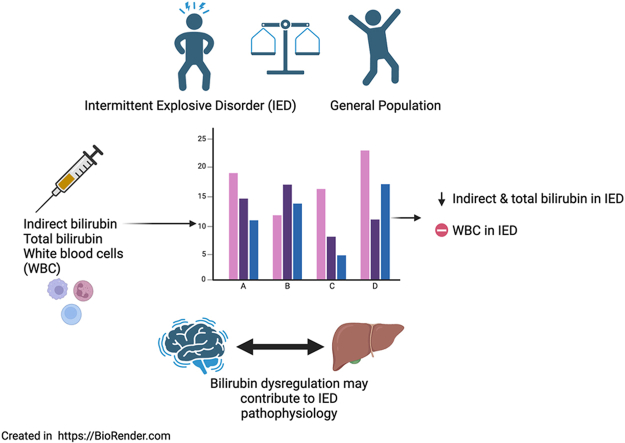

## 1 Introduction

Intermittent Explosive Disorder (IED) is a psychiatric condition defined by recurrent episodes of impulsive aggression and disproportionate anger responses. Estimates of its prevalence vary across studies and populations, with reported rates generally ranging from 1 % to 7 % in community samples [1]. IED is associated with functional impairment, comorbid psychiatric conditions, and adverse long-term health outcomes. While considerable research has advanced our understanding of the neural correlates of IED—highlighting alterations in limbic reactivity, serotonergic signaling, and stress-regulatory systems [[2], [3], [4]]—there remains a limited focus on systemic metabolic processes that may contribute to the disorder's pathophysiology.

Emerging evidence across psychiatric disorders supports a broader biological framework in which systemic inflammation and oxidative stress contribute to mental health vulnerability. In IED, several studies have identified elevated levels of inflammatory markers and oxidative stress biomarkers—such as C-reactive protein (CRP) and interleukin-6 (IL-6)—which have been associated with aggression severity and early life adversity [[5], [6], [7], [8]]. These findings point to a possible role of peripheral immune activation and stress-related physiological alterations in the disorder's pathophysiology. The heterogeneity of IED presentations, including variation in comorbidities and environmental exposures, may give rise to distinct inflammatory or metabolic signatures across individuals. While most IED research has focused on central nervous system mechanisms, there is growing recognition that peripheral systems—such as immune and hepatic function—may also influence behavioral dysregulation [9]. Notably, the contribution of hepatobiliary processes, including bilirubin metabolism, has not been systematically investigated in IED. This gap highlights the need to explore how broader inflammatory and metabolic pathways may intersect with impulsive aggression in this population.

Bilirubin, a byproduct of heme catabolism, has traditionally been viewed through the lens of liver function testing, but more recently, it has been recognized as a potent endogenous antioxidant with important anti-inflammatory properties [10]. Indirect (unconjugated) bilirubin in particular plays a key role in countering oxidative stress and neutralizing free radicals [11,12]. Alterations in bilirubin metabolism may therefore reflect underlying disturbances in redox homeostasis or hepatic processing, especially in states characterized by chronic stress or inflammation. While benign elevations in unconjugated bilirubin, such as in Gilbert's syndrome [13], have not been directly associated with psychiatric pathology, they highlight how changes in enzymatic conjugation pathways and hepatic metabolism can meaningfully alter circulating bilirubin profiles in otherwise healthy individuals. Furthermore, stress-induced changes in liver function and systemic inflammation have been shown to impact bilirubin production and clearance [14], reinforcing the need to investigate bilirubin metabolism more broadly in psychiatric populations.

This study addresses a critical gap in the literature by being the first to examine bilirubin profiles in individuals with IED, integrating perspectives from hepatobiliary physiology, inflammatory signaling, and oxidative stress. Drawing on insights from both psychiatric and metabolic research, we propose that several converging mechanisms may contribute to altered bilirubin metabolism in IED. These include increased oxidative stress [11,12] leading to greater antioxidant consumption; sympathetic nervous system overactivation [[2], [3], [4]] influencing hepatic hemodynamics and metabolic processing; subtle impairments in hepatic conjugation or biliary clearance [15]; possible trait-level differences in heme oxygenase or UGT1A1 activity [16]; and behavioral patterns associated with IED, such as erratic eating, substance use, or poor nutritional intake, that may disrupt hepatic function [17].

In this exploratory and preliminary study, we examined indirect bilirubin, total bilirubin, and white blood cell (WBC) count among individuals diagnosed with IED, comparing their profiles to those of a matched general population (GP) control group. We hypothesized that individuals with IED would exhibit lower levels of circulating indirect bilirubin, reflecting diminished antioxidant capacity in the context of chronic oxidative stress, and elevated WBC count, consistent with heightened systemic inflammatory burden. By investigating these metabolic and immune signatures, our goal is to begin clarifying the physiological correlates of IED and to generate new hypotheses for how metabolic, inflammatory, and psychiatric processes intersect in the disorder's etiology and progression.

## 2 Materials and methods

This study analyzed laboratory data from individuals diagnosed with IED and a GP comparison group (Table 1). Participants were drawn from a large clinical research database, All of Us Research Program version 7 [18], and included only those with available biliary function panel data and white blood cell (WBC) counts. Eligibility for inclusion in the IED group required a confirmed clinical diagnosis based on SNOMED codes extracted from electronic health records across outpatient and inpatient settings. The GP comparison group comprised individuals with no documented history of IED. To reduce potential confounding, participants were excluded if they had a documented history of liver diseases—including hepatitis (viral, autoimmune, or alcoholic), cirrhosis, liver tumors, or non-alcoholic fatty liver disease—as well as hemolytic disorders such as hemolytic anemia, sickle cell disease, thalassemia, glucose-6-phosphate dehydrogenase (G6PD) deficiency, or prior transfusion or drug-induced hemolytic reactions. Biliary tract conditions such as gallstones, cholangitis, biliary atresia, primary sclerosing cholangitis, or primary biliary cholangitis, and rare genetic disorders affecting bilirubin metabolism, including Crigler-Najjar syndrome and Dubin-Johnson syndrome, were also grounds for exclusion. Additional exclusions included individuals with autoimmune or inflammatory disorders such as rheumatoid arthritis, systemic lupus erythematosus (SLE), inflammatory bowel disease, psoriasis, ankylosing spondylitis, sarcoidosis, or systemic vasculitis. Participants with evidence of acute or chronic infections, including tuberculosis, HIV, or sepsis, as well as those with malignancies such as lymphoma, leukemia, or actively progressing solid tumors, were also excluded. Finally, individuals with other chronic conditions known to affect systemic inflammation, including polycystic ovary syndrome (PCOS) with metabolic features, chronic obstructive pulmonary disease (COPD), pancreatitis, and bronchitis, were not included in the analysis.

**Table 1:** Participant demographics and matching statistics.

Variable	GP	IED	Effect Size	SMD Before Matching	SMD After Matching
Age (years)	45.50	44.70	0.06	−1.15	−0.06
BMI (kg/m^2^)	31.10	31.20	−0.01	0.26	0.01
Annual Alcohol Frequency∗	2.08	2.16	−0.07	−0.09	0.07
Smoking Frequency∗	1.12	1.12	−0.01	0.48	0.01
Binge Frequency∗	1.62	1.59	0.02	0.55	−0.02
Avg Drinks∗	1.80	1.78	0.02	0.26	−0.02
Sex: Female (%)	37.50	40.60	0.00	−0.08	0.03
Sex: Prefer not to answer or skipped (%)	3.40	3.10	0.00	0.01	0.00
Race: Black or African American (%)	32.50	31.20	0.00	0.05	−0.01
Race: More than one population (%)	5.60	6.20	0.00	0.04	0.01
Race: None Indicated (%)	6.60	6.20	0.00	−0.05	0.00
Race: None of these (%)	2.20	3.10	0.00	0.02	0.01
Race: Skip (%)	7.80	6.20	0.00	0.04	−0.02
Race: White (%)	45.30	46.90	0.00	−0.08	0.02
Ethnicity: Hispanic or Latino (%)	13.80	12.50	0.00	−0.01	−0.01
Ethnicity: Not Hispanic or Latino (%)	76.20	78.10	0.00	−0.05	0.02
Ethnicity: Skip (%)	7.80	6.20	0.00	0.04	−0.02
Ethnicity: What Race Ethnicity: Race Ethnicity None of These (%)	2.20	3.10	0.00	0.02	0.01

***Caption****:* Participant demographics and standardized mean differences (SMDs) before and after propensity score matching for the General Population (GP) and Intermittent Explosive Disorder (IED) groups. The table includes means or percentages for each group, effect sizes (Cohen's *d* for continuous variables, Cramer's *V* for categorical variables), and SMDs to assess covariate balance pre- and post-matching. The final analytic sample consisted of 320 participants in the GP group and 32 participants in the IED group. Matching was effective, with all post-matching SMDs near zero, indicating excellent covariate balance between groups. Skip indicates a participant skipped the question.

*∗Annual alcohol frequency* was measured on a scale from 0 (Never) to 4 (4 or more times per week), based on self-reported past-year drinking frequency.

**∗***Smoking frequency* was measured on a scale from 0 (Not at all) to 2 (Every day), based on self-reported current smoking behavior.

**∗***Binge frequency* was measured on a scale from 0 (Never in the past year) to 4 (Daily), based on self-reported frequency of consuming six or more drinks on one occasion during the past year.

**∗***Average drinks* was measured on a scale from 1 (1–2 drinks per day) to 5 (10 or more drinks per day), based on self-reported average daily alcohol consumption.

The study was conducted in accordance with the Code of Ethics of the World Medical Association (Declaration of Helsinki) for experiments involving humans. Informed consent was obtained from all participants, and the privacy rights of participants were strictly observed for completion of the study.

### 2.1 Laboratory data collection

Laboratory data collected from outpatient and inpatient settings included alanine aminotransferase (ALT), aspartate aminotransferase (AST), total bilirubin, indirect (unconjugated) bilirubin, and white blood cell (WBC) count. These biomarkers were selected to assess liver function, bilirubin metabolism, and systemic inflammation. Not all participants had all biomarkers evaluated; those with incomplete laboratory data were excluded from analyses requiring complete data (e.g., group comparisons but not linear mixed effects models). Both raw values and summary statistics, including mean and standard deviation, were computed for all biomarkers.

#### 2.1.1 Propensity score matching

To reduce potential confounding and improve the comparability between the IED and GP groups, 10:1 propensity score matching (PSM) was conducted [19]. Propensity scores were estimated using a logistic regression model that included the following covariates: self-reported age (continuous, in years), sex (binary), race (categorical), ethnicity (binary), annual alcohol frequency (ordinal), smoking frequency (ordinal), binge drinking frequency (ordinal), average number of drinks per drinking day (continuous), and measured body mass index (BMI, continuous).

Annual alcohol frequency was assessed using the prompt, “How often did you have a drink containing alcohol in the past year?” and coded on a 5-point scale: Never (1), Monthly or less (2), 2 to 4 times per month (3), 2 to 3 times per week (4), and 4 or more times per week (5). Smoking frequency and binge drinking frequency were assessed using five-level ordinal scales derived from structured questionnaire items.

Binge drinking frequency was assessed using the item: *“In the past year, how often did you drink five or more drinks of alcohol in a row, within a couple of hours?”* with similar response coding from 0 to 4, where higher values reflected more frequent binge episodes. Average number of drinks per drinking day was treated as a continuous variable and derived from responses to two items: one asking about the typical number of drinks per occasion, and another about the frequency of drinking days.

Smoking frequency was measured by the item: *“How often do you currently smoke tobacco products (*e.g.*, cigarettes, cigars, pipe, hookah)?"* with response options: (0) Never, (1) Less than once a month, (2) 1–3 times a month, (3) 1–6 times a week, and (4) Daily or almost daily.

Nearest neighbor propensity score matching without replacement was applied using a 1:10 ratio (IED to GP), ensuring balance across demographic variables (age, sex, race, ethnicity, BMI) and behavioral covariates (alcohol frequency, smoking frequency, binge frequency, and average number of drinks per drinking day).

We evaluated the performance of the propensity score model by examining standardized mean differences (SMDs) before and after matching (Table 1), with values below 0.1 indicating adequate covariate balance. Visual inspection of covariate distributions and overlapping propensity score densities confirmed good balance between groups. A caliper was not applied, as sufficient overlap was observed without it.

Propensity score matching (PSM) [19] was selected to strengthen causal inference by reducing observed confounding and improving group comparability. This approach allowed for the creation of a demographically and behaviorally balanced subsample for group comparisons. PSM was favored over traditional regression adjustment alone to better approximate the conditions of a randomized controlled trial, enhancing the validity of comparisons by minimizing bias due to measured baseline differences.

#### 2.1.2 Sensitivity analyses

To assess the robustness of group-level associations to potential unmeasured confounding, we conducted sensitivity analyses [20] using the sensemakr R package [21]. Linear models were specified separately for each biomarker (indirect bilirubin, total bilirubin, and WBC), including fixed effects for group (IED vs. GP), age, gender, race, ethnicity, BMI, alcohol frequency, smoking frequency, binge drinking frequency, and average daily alcohol consumption. The primary treatment variable was group status (IED vs. GP). For each model, we estimated the robustness value, defined as the minimum strength that an unmeasured confounder would need (in terms of its partial R^2^ with both the treatment and the outcome) to fully explain away the observed group effect. Benchmarks for comparison included the explanatory power of observed covariates such as age and BMI. Sensitivity contour plots were generated to visually depict the range of unmeasured confounding required to attenuate the observed effects to zero or render them statistically nonsignificant (Supplementary Fig. 1).

### 2.2 Statistical analysis

Group comparisons were conducted to evaluate differences in biomarker levels between the IED and GP groups using a matched dataset derived from propensity score matching. For each unique participant, laboratory values were averaged across all available clinical visits—including both outpatient and inpatient encounters—to generate a single mean value per biomarker. This averaging approach accounted for within-subject variability and was designed to capture stable, trait-like physiological characteristics.

Group comparisons were performed using Welch's two-sample t-tests [22] to accommodate unequal variances, with statistical significance defined as p < 0.05. All p-values reported for group comparisons of biomarker levels are unadjusted. As none of the results reached statistical significance at the α = 0.05 level, no additional multiple comparison correction (e.g., Bonferroni [23] or FDR [24]) was applied, as this would not change the interpretation. Cohen's d was used to quantify the magnitude of observed differences, with benchmarks for interpretation as negligible (<0.2), small (0.2–0.49), medium (0.5–0.79), or large (≥0.8) effects [25].

### 2.3 Linear mixed-effects modeling

Linear mixed-effects models (LMMs) [26,27] were employed to analyze repeated laboratory measurements and account for within-subject variability, as many participants contributed multiple biomarker values across different clinical visits. Hepatic function biomarkers—including total bilirubin, indirect bilirubin, and white blood cell (WBC) count—were modeled as dependent variables, with age, sex, race, ethnicity, body mass index (BMI), annual alcohol use frequency, smoking frequency, binge drinking frequency, and average number of drinks per drinking day included as fixed-effect covariates.

Participant ID was included as a random intercept to account for the nested structure of the data and to model the correlation of repeated measures within individuals. A random intercept model was selected based on the study's focus on average differences between groups while accounting for within-person clustering, without assuming subject-specific variation in the effects of covariates. Alternative model structures, including random slopes for time-varying covariates and visit order, were explored but did not substantially improve model fit as evaluated by Akaike Information Criterion (AIC) [28] and likelihood ratio tests [29]. Moreover, including random slopes led to convergence issues in some cases, likely due to limited within-subject variability in covariates such as BMI or alcohol use frequency. Each linear mixed-effects model demonstrated reasonable fit to the data. Marginal R^2^ values reflected the proportion of variance explained by fixed effects alone, while conditional R^2^ values captured the combined variance explained by both fixed and random effects (i.e., including subject-level random intercepts).

Model diagnostics were performed to assess residual distributions, homoscedasticity, and linearity assumptions (Supplementary Fig. 2). Residuals vs. fitted value plots for total and indirect bilirubin suggested no major violations of homoscedasticity or linearity. Q-Q plots for these biomarkers showed generally approximate normality of residuals, with slight deviations at the tails. In contrast, residual diagnostics for WBC revealed greater dispersion and more pronounced non-normality, with heavy-tailed deviations in the Q-Q plot. Taken together, these diagnostics support the adequacy of the random intercept model for bilirubin outcomes, while suggesting greater heterogeneity and model misfit for WBC, consistent with its weaker and more confounded association in sensitivity analyses.

All statistical analyses were performed using R (version 4.2.2) [30]. Data manipulation was conducted using the dplyr package [31], while visualizations were generated using ggplot2 [32]. Propensity score matching was performed using the MatchIt package [33], and linear mixed-effects models were fitted using lme4 [34]. Effect sizes were computed using the effectsize package [35], and statistical tests were carried out using the built-in stats package. Group differences in biomarker levels—including total bilirubin, indirect bilirubin, and white blood cell (WBC) count—were visualized to facilitate interpretation of results.

## 3 Results

**Fig. 1:**
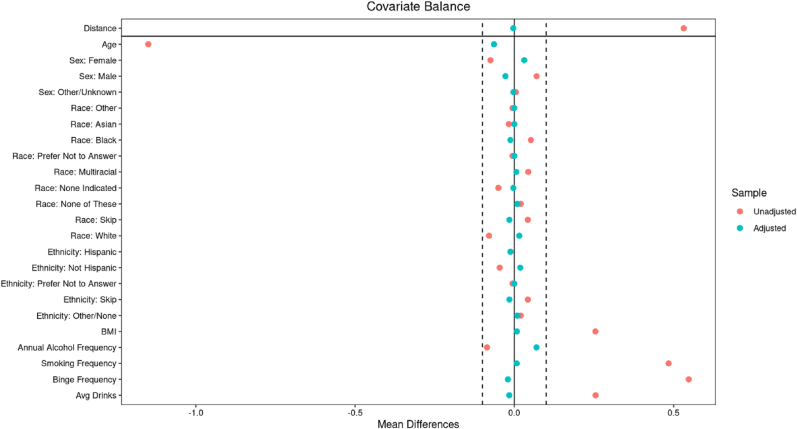
Covariate Balance Before and After Matching **Caption:** This figure displays the standardized mean differences for key covariates between the Intermittent Explosive Disorder (IED) and General Population (GP) groups, before and after 10:1 nearest-neighbor propensity score matching. Variables include demographic factors (e.g., age, sex, race, ethnicity) and clinical indicators (e.g., BMI, annual alcohol frequency, smoking frequency, binge frequency, and average drinks per drinking day). Red dots represent unadjusted differences, while blue dots represent adjusted differences. After matching, most covariates fall within the ±0.1 threshold (dashed lines), indicating good covariate balance and minimizing potential confounding in subsequent group comparisons.

Propensity score matching using a 10:1 nearest-neighbor procedure successfully balanced key demographic (age, sex, race, ethnicity) and clinical (BMI, annual alcohol frequency, smoking frequency, binge drinking frequency, and average number of drinks per drinking day) covariates between the IED and GP groups. As shown in Table 1, the matched groups were comparable across all covariates, with no significant differences in sex distribution, mean age, race, ethnicity, BMI, or substance use variables (p > 0.05 for all). Covariate balance diagnostics demonstrated that standardized mean differences after matching clustered closely around zero, indicating strong overlap and minimal residual bias (Fig. 1). This balance ensures that subsequent analyses involving biomarker data are not confounded by baseline covariate differences.

**Fig. 2:**
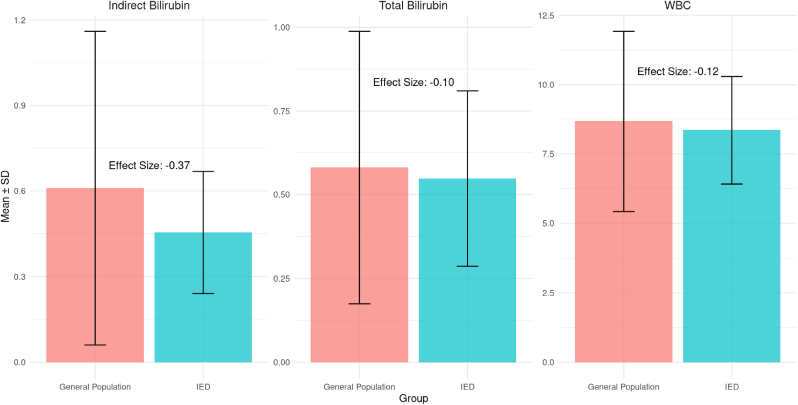
Group Differences in Bilirubin and WBC Levels Using Matched Sample **Caption:** This figure displays the mean levels (± standard deviation) of indirect bilirubin, total bilirubin, and white blood cell (WBC) count in individuals with Intermittent Explosive Disorder (IED) compared to a matched General Population (GP) sample. Matching was performed using demographic and behavioral covariates including age, sex, race, ethnicity, BMI, alcohol frequency, smoking frequency, binge drinking frequency, and average number of drinks per drinking day. No additional weighting or propensity score adjustment was applied. Effect sizes (Cohen's *d*) are shown for each biomarker. Results reveal moderately lower levels of indirect bilirubin (effect size = −0.37) and slightly lower total bilirubin (effect size = −0.10) in the IED group, with minimal differences in WBC (effect size = −0.12). These findings suggest potential differences in bilirubin metabolism in IED, with less evidence for elevated systemic inflammation as reflected by WBC levels.

Group-level comparisons of biomarker profiles were conducted using a matched sample of individuals with IED and GP controls identified through 10:1 nearest-neighbor propensity score matching. Mean values and standard deviations for indirect bilirubin, total bilirubin, and white blood cell (WBC) count are shown in Fig. 2. Compared to the matched GP group, individuals with IED exhibited lower levels of both indirect and total bilirubin. The effect size for indirect bilirubin was moderate (d = −0.37), while the effect size for total bilirubin was smaller (d = −0.10). WBC count was also slightly lower in the IED group, with a small effect size (d = −0.12), suggesting minimal group differences in systemic inflammation as indexed by this marker. These findings point to subtle but potentially meaningful alterations in bilirubin metabolism among individuals with IED.

**Fig. 3:**
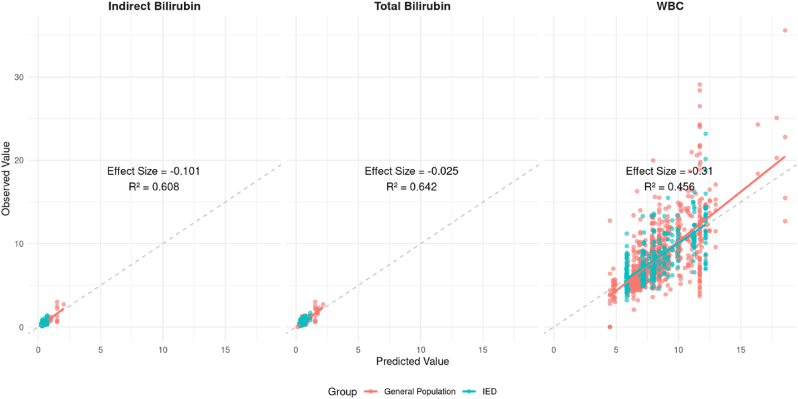
Predicted vs. Observed Individual Bilirubin Levels (Linear Mixed Effects Model) **Caption:** This figure displays the relationship between predicted and observed values for indirect bilirubin, total bilirubin, and white blood cell (WBC) count, based on linear mixed-effects models adjusted for age, sex, race, ethnicity, BMI, annual alcohol frequency, binge drinking frequency, smoking frequency, and average number of drinks per drinking day. Participant ID was included as a random intercept to account for repeated measures. Each panel shows the effect size for IED vs. GP group status, as well as model fit statistics. For **indirect bilirubin**, the group effect was modestly negative (effect size = −0.101), with a marginal R^2^ of 0.316 (variance explained by fixed effects) and a conditional R^2^ of 0.653 (variance explained by fixed and random effects). **Total bilirubin** showed a weaker group association (effect size = −0.025), but the model still demonstrated good overall fit, with a marginal R^2^ of 0.113 and conditional R^2^ of 0.735. In contrast, **WBC** exhibited a larger negative group effect (effect size = −0.310), but with poorer model fit (marginal R^2^ = 0.144; conditional R^2^ = 0.483), reflecting greater heterogeneity in the data. Sensitivity analyses indicated that the WBC finding was less robust to unmeasured confounding. These results support consistent, modest reductions in bilirubin levels among individuals with IED, while evidence for group differences in WBC remains more uncertain.

To further assess the consistency and precision of group-level differences in biomarker values, linear mixed-effects models (LMMs) were fit using all available repeated measurements for each participant. Each model included fixed effects for IED status and covariates including age, sex, race, ethnicity, BMI, alcohol frequency, binge drinking frequency, smoking frequency, and average drinks per drinking day, with a random intercept for participant ID. As shown in Fig. 3, indirect bilirubin exhibited a small group difference (effect size = −0.101), with strong model fit (R^2^ = 0.608). Total bilirubin demonstrated an even smaller effect (−0.025) with slightly better fit (R^2^ = 0.642). In contrast, WBC showed a more pronounced group effect (−0.310), but with lower explanatory power (R^2^ = 0.456), reflecting greater variability in WBC values. Sensitivity analyses presented in Supplementary Fig. 1 suggest that the observed bilirubin effects were relatively robust to potential unmeasured confounding, whereas the WBC association was more vulnerable to bias and not statistically robust. These findings support modest and consistent reductions in bilirubin in the IED group, while WBC differences were less stable.

While effect sizes suggested potentially meaningful group differences in biomarker levels between the IED and GP groups, all statistical tests yielded non-significant p-values after adjusting for covariates in the linear mixed-effects (LME) models. Specifically, p-values for group status (IED vs. GP) were 0.423 for indirect bilirubin, 0.729 for total bilirubin, and 0.585 for white blood cell (WBC) count. These results indicate that, despite observed trends—such as lower indirect bilirubin levels in the IED group—the differences were not statistically robust. The lack of significance is likely attributable to limited power due to the relatively small sample size in the IED group, as well as natural variability in these physiological markers.

Similarly, when group comparisons were repeated using unadjusted means from the matched sample, the pattern of non-significance persisted. Indirect bilirubin showed a modest effect size but a non-significant p-value of 0.237, while total bilirubin and WBC yielded p-values of 0.607 and 0.518, respectively. These results further reinforce that none of the group differences met the conventional threshold for statistical significance (α = 0.05), and would not reach significance even if multiple comparison corrections (e.g., Bonferroni or FDR) were applied. Collectively, these findings suggest that while the directionality and consistency of bilirubin reductions in the IED group are noteworthy, they should be interpreted with caution due to statistical uncertainty, and that WBC is likely too variable to serve as a reliable biomarker in this context.

## 4 Discussion

This study investigated bilirubin metabolism and systemic biomarkers in individuals with IED compared to a GP control group, using a matched sample and linear mixed-effects modeling. We found modest but consistent differences in bilirubin profiles—specifically, reduced levels of indirect and total bilirubin in the IED group—alongside minimal differences in white blood cell (WBC) counts. These results suggest that individuals with IED may exhibit a distinct peripheral metabolic profile involving bilirubin regulation. However, the role of systemic inflammation could not be fully assessed in this study due to limited availability of data for key inflammatory markers such as C-reactive protein (CRP) and interleukin-6 (IL-6).

### 4.1 Comparison to prior literature

Previous studies have reported elevated levels of CRP, IL-6, and other inflammatory markers in individuals with IED [5,6], often linking these elevations to greater aggression severity and histories of early life adversity [5,8]. While our findings did not show marked group-level differences in WBC count, the lack of available data on CRP and IL-6 limits our ability to draw conclusions about systemic inflammation in this sample. It remains possible that inflammatory processes play a role in IED pathophysiology, but further work with more complete inflammatory biomarker data is needed to clarify this relationship.

Rather than contradicting prior findings, our results may reflect the underlying heterogeneity in physiological mechanisms across individuals with IED. The consistent reduction in bilirubin—an endogenous antioxidant and metabolic byproduct—may signal an alternative or complementary biological pathway related to oxidative stress and metabolic vulnerability. This shift away from a purely inflammatory model highlights the potential involvement of hepatobiliary function and redox balance in the neurobiological underpinnings of impulsive aggression.

### 4.2 Potential mechanisms for altered bilirubin in IED

The observed reduction in indirect and total bilirubin levels in individuals with IED may reflect several overlapping physiological mechanisms. One possibility is that chronic oxidative stress in IED depletes circulating unconjugated bilirubin, a potent endogenous antioxidant [10], resulting in reduced systemic antioxidant capacity. Another mechanism may involve sympathetic nervous system overactivation [[2], [3], [4]]—well-documented in IED—which can influence hepatic perfusion and bilirubin clearance, potentially accelerating hepatic uptake and conjugation of bilirubin. A third explanation may involve subclinical hepatobiliary dysfunction, including subtle changes in conjugation or excretion processes [15], which could lead to altered serum bilirubin profiles even in the absence of overt liver disease.

In addition, trait-level differences in enzymatic pathways such as heme oxygenase activity or UGT1A1 function [16] may influence baseline bilirubin levels in individuals with IED. Finally, behavioral and lifestyle patterns commonly associated with IED—such as erratic eating, impulsive substance use, or poor nutritional intake [17]—could compromise liver function or reduce the availability of bilirubin precursors, further contributing to the observed patterns.

Given the limited availability of inflammatory biomarker data in this sample, the hepatobiliary explanation for reduced bilirubin levels emerges as a compelling potential pathway. While systemic inflammation and oxidative stress remain relevant frameworks based on prior literature, the current findings favor a physiological model in which bilirubin metabolism may be altered through hepatic or broader metabolic dysregulation. Without sufficient CRP or IL-6 data, we cannot rule out an inflammatory contribution; however, the observed patterns point toward non-inflammatory mechanisms—particularly those involving liver function and redox balance—as likely contributors to bilirubin alterations in individuals with IED.

### 4.3 Implications and future directions

These findings suggest that bilirubin metabolism may represent a novel and underrecognized pathway in the pathophysiology of IED. If validated in larger and more comprehensively phenotyped samples, bilirubin-related biomarkers could hold potential for stratifying patients or identifying individuals who may benefit from metabolic or antioxidant-based interventions. While inflammatory processes have been implicated in prior research, the current study lacked sufficient data on key markers such as CRP and IL-6 to fully evaluate this pathway. As such, the absence of strong inflammatory signal in our data should be interpreted with caution and reinforces the importance of targeted biomarker selection and adequate sampling in future investigations.

Moving forward, research should incorporate more granular measures of liver function and explore specific enzymes involved in heme metabolism and bilirubin conjugation. Longitudinal designs will be critical to determining whether bilirubin alterations precede IED onset or emerge as a consequence of chronic stress and dysregulation. Interventions aimed at modulating hepatic function or boosting antioxidant capacity [[36], [37], [38], [39], [40]] may also hold promise as adjunctive treatment strategies for individuals with IED.

### 4.4 Limitations

This study has several important limitations. First, bilirubin metabolism is influenced by a complex interplay of genetic, environmental, and behavioral factors. Without genotyping or more detailed clinical characterization, it is difficult to determine the extent to which genetic variants (e.g., UGT1A1 polymorphisms associated with Gilbert's syndrome [13]) contributed to the observed group differences. While individuals with known liver disease were excluded, we could not rule out the presence of benign Gilbert's syndrome, which may have influenced baseline bilirubin levels.

Second, the GP comparison group may not fully reflect a “healthy” control population matched to IED patients in terms of lifestyle or socioeconomic background. We lacked key lifestyle data such as income and physical activity (e.g., Fitbit metrics) patterns in the IED group, limiting our ability to match on these variables or to isolate lifestyle-driven effects. This introduces the potential for residual confounding based on unmeasured social determinants of health.

Third, we were unable to assess inflammatory markers such as IL-6 or CRP in this sample due to data limitations (i.e., small sample size), precluding a more comprehensive evaluation of inflammatory processes. Similarly, potential confounding by medications known to influence hepatic metabolism, such as over-the-counter supplements or agents like St. John's Wort, could not be evaluated, though this represents a critical avenue for future work.

Fourth, the statistical methods used—propensity score matching and linear mixed-effects models—have their own assumptions and constraints. Propensity scores rely on correctly specified models, and if important covariates were omitted or mis specified, bias may remain. Linear mixed-effects models assume linear relationships between covariates and outcomes, which may oversimplify complex biological interactions. Additionally, sample size limitations prevented us from separating healthy and psychiatric controls within the GP group, which could have helped further isolate IED-specific effects.

Fifth, while some biomarkers—particularly indirect bilirubin—showed small-to-moderate effect sizes, the group differences were not statistically significant. This discrepancy is likely due in part to the limited sample size of the IED group, which reduced statistical power and increased uncertainty in model estimates. As a result, potentially meaningful physiological differences may have gone undetected under conventional significance thresholds. These findings should therefore be interpreted as preliminary and hypothesis-generating, rather than conclusive.

Despite these limitations, the use of repeated measures, matched sampling, and multiple analytic strategies strengthens confidence in the overall pattern of findings and provides a foundation for more targeted investigations into the metabolic underpinnings of IED.

## 5 Conclusions

This study provides preliminary evidence of altered bilirubin metabolism in individuals with IED, characterized by consistent reductions in both indirect and total bilirubin compared to a matched GP group. While prior research has emphasized systemic inflammation in IED, our findings—limited by insufficient data on key inflammatory markers such as CRP and IL-6—highlight the potential relevance of hepatobiliary and metabolic pathways in this population. By pointing to disruptions in bilirubin-related antioxidant processes, the current study broadens existing models of IED to include systemic physiological mechanisms and underscores the need for future research integrating metabolic, inflammatory, and behavioral dimensions of the disorder.

## CRediT authorship contribution statement

**Jeffrey B. Schwimmer:** Writing – review & editing, Methodology, Conceptualization. **Murray B. Stein:** Writing – review & editing, Visualization, Methodology. **Emil F. Coccaro:** Writing – review & editing, Methodology. **Alejandro D. Meruelo:** Writing – review & editing, Writing – original draft, Visualization, Funding acquisition, Formal analysis, Conceptualization.

## Data statement

This work utilized data from the All of Us Research Program (https://allofus.nih.gov), which is funded by the National Institutes of Health. The data analyzed in this research are accessible to authorized researchers through the All of Us Research Hub, in accordance with program data access policies.

## Funding sources

This research was supported by the 10.13039/100000027National Institute on Alcohol Abuse and Alcoholism (10.13039/100000027NIAAA) under grant K23 AA026869 awarded to Alejandro Meruelo, MD, PhD.

## Declaration of competing interest

Dr. Jeffrey B. Schwimmer is a current recipient of grant funding from the 10.13039/100000062National Institute of Diabetes and Digestive and Kidney Diseases (10.13039/100000062NIDDK). Over the past three years, Dr. Murray B. Stein has received consulting fees from multiple organizations, including Aptinyx, atai Life Sciences, BigHealth, Biogen, Bionomics, Boehringer Ingelheim, Delix Therapeutics, EmpowerPharm, Engrail Therapeutics, Janssen, Jazz Pharmaceuticals, Karuna Therapeutics, NeuroTrauma Sciences, Otsuka US, PureTech Health, Sage Therapeutics, and Roche/Genentech. He holds stock options in Oxeia Biopharmaceuticals and EpiVario. Additionally, he has been compensated for editorial roles, serving as Editor-in-Chief for *Depression and Anxiety*, Deputy Editor for *Biological Psychiatry*, and Co-Editor-in-Chief for Psychiatry at UpToDate. Dr. Stein has also received research funding from the 10.13039/100000002NIH, the 10.13039/100000738Department of Veterans Affairs, and the 10.13039/100000005Department of Defense. Furthermore, he serves on the scientific advisory boards of the Brain and Behavior Research Foundation and the Anxiety and Depression Association of America. Dr. Emil F. Coccaro serves as a consultant and member of the Scientific Advisory Boards for Azevan Pharmaceuticals, Inc., Avanir Pharmaceuticals, Inc., and Boehringer Ingelheim Pharma, Inc. He is also a current recipient of grant funding from the 10.13039/100000025National Institute of Mental Health (10.13039/100000025NIMH) and the 10.13039/100000027National Institute on Alcohol Abuse and Alcoholism (10.13039/100000027NIAAA). Dr. Alejandro D. Meruelo is a current recipient of grant funding from the 10.13039/100000027National Institute on Alcohol Abuse and Alcoholism (10.13039/100000027NIAAA).

## References

[1] Coccaro E.F., Posternak M.A., Zimmerman M. (2005). Prevalence and features of intermittent explosive disorder in a clinical setting. J. Clin. Psychiatry.

[2] Gollan J.K., Lee R., Coccaro E.F. (2005). Developmental psychopathology and neurobiology of aggression. Dev. Psychopathol..

[3] Mbiydzenyuy N.E., Qulu L.-A. (2024). Stress, hypothalamic-pituitary-adrenal axis, hypothalamic-pituitary-gonadal axis, and aggression. Metab. Brain Dis..

[4] Rosell D.R., Siever L.J. (2015). The neurobiology of aggression and violence. CNS Spectr..

[5] Coccaro E.F., Lee R., Coussons-Read M. (2014). Elevated plasma inflammatory markers in individuals with intermittent explosive disorder and correlation with aggression in humans. JAMA Psychiatry.

[6] Coccaro E.F., Lee R., Gozal D. (2016). Elevated plasma oxidative stress markers in individuals with intermittent explosive disorder and correlation with aggression in humans. Biol. Psychiatry.

[7] Coccaro E.F., Lee R., Breen E.C., Irwin M.R. (2023). Plasma and cerebrospinal fluid inflammatory markers and human aggression. Neuropsychopharmacology.

[8] Fanning J.R., Lee R., Gozal D., Coussons-Read M., Coccaro E.F. (2015). Childhood trauma and parental style: relationship with markers of inflammation, oxidative stress, and aggression in healthy and personality disordered subjects. Biol. Psychol..

[9] Azhari H., Swain M.G. (2018). Role of peripheral inflammation in hepatic encephalopathy. J Clin Exp Hepatol.

[10] Ramírez-Mejía M.M., Castillo-Castañeda S.M., Pal S.C., Qi X., Méndez-Sánchez N. (2024). The multifaceted role of bilirubin in liver disease: a literature review. J Clin Transl Hepatol.

[11] Sandoval H.O.G. (2009).

[12] Punzo A., Silla A., Fogacci F., Perillo M., Cicero A.F.G., Caliceti C. (2024). Bile acids and bilirubin role in oxidative stress and inflammation in cardiovascular diseases. Dis Basel Switz.

[13] Wagner K.-H., Shiels R.G., Lang C.A., Seyed Khoei N., Bulmer A.C. (2018). Diagnostic criteria and contributors to Gilbert's syndrome. Crit. Rev. Clin. Lab Sci..

[14] Yamaguchi T., Shioji I., Sugimoto A., Yamaoka M. (2002). Psychological stress increases bilirubin metabolites in human urine. Biochem. Biophys. Res. Commun..

[15] Memon N., Weinberger B.I., Hegyi T., Aleksunes L.M. (2016). Inherited disorders of bilirubin clearance. Pediatr. Res..

[16] Petrtýl J., Dvořák K., Stříteský J., Leníček M., Jirásková A., Šmíd V. (2021). Association of serum bilirubin and functional variants of heme oxygenase 1 and bilirubin UDP-glucuronosyl transferase genes in Czech adult patients with non-alcoholic fatty liver disease. Antioxidants.

[17] Tarantino G., Cataldi M., Citro V. (2022). Could alcohol Abuse and dependence on junk foods inducing obesity and/or illicit drug use represent danger to liver in young people with altered psychological/relational spheres or emotional problems?. Int. J. Mol. Sci..

[18] (2019). The “all of us” research program. N. Engl. J. Med..

[19] Austin P.C. (2011). An introduction to propensity score methods for reducing the effects of confounding in observational studies. Multivariate Behav. Res..

[20] Cinelli C., Hazlett C. (2020). Making sense of sensitivity: extending omitted variable bias. J R Stat Soc Ser B Stat Methodol.

[21] Cinelli C., Ferwerda J., Hazlett C., Tsao D., Rudkin A., Ljubownikow G. (2024).

[22] West R.M. (2021). Best practice in statistics: use the Welch t-test when testing the difference between two groups. Ann. Clin. Biochem..

[23] Armstrong R.A. (2014). When to use the Bonferroni correction. Ophthalmic Physiol. Opt..

[24] Noble W.S. (2009). How does multiple testing correction work?. Nat. Biotechnol..

[25] Cohen J. (1988). Statistical Power Analysis for the Behavioral Sciences.

[26] Gałecki A., Burzykowski T., Gałecki A., Burzykowski T. (2013). Linear Mix.-Eff. Models Using R Step-sStep Approach.

[27] Bates D., Mächler M., Bolker B., Walker S. (2015). Fitting linear mixed-effects models using lme4. J. Stat. Software.

[28] Akaike H. (1998). Information theory and an extension of the maximum likelihood principle. Sel. Pap. Hirotugu Akaike.

[29] Lewis F., Butler A., Gilbert L. (2011). A unified approach to model selection using the likelihood ratio test. Methods Ecol. Evol..

[30] R Core Team (2021).

[31] Wickham H., François R., Henry L., Müller K., Vaughan D., Software P. (2023).

[32] Wickham H. (2016).

[33] Ho DE, Imai K, King G, Stuart EA. MatchIt: Nonparametric Preprocessing for Parametric Causal Inference n.d.

[34] Fitting Linear Mixed-Effects Models Using lme4 | J. Stat. Software n.d. https://www.jstatsoft.org/article/view/v067i01 (accessed November 24, 2024).

[35] Ben-Shachar M.S., Lüdecke D., Makowski D. (2020). Effectsize: estimation of effect size indices and standardized parameters. J. Open Source Softw..

[36] Minarini A., Ferrari S., Galletti M., Giambalvo N., Perrone D., Rioli G. (2017). N-acetylcysteine in the treatment of psychiatric disorders: current status and future prospects. Expet Opin. Drug Metabol. Toxicol..

[37] Lee T.-M., Lee K.-M., Lee C.-Y., Lee H.-C., Tam K.-W., Loh E.-W. (2021). Effectiveness of N-acetylcysteine in autism spectrum disorders: a meta-analysis of randomized controlled trials. Aust. N. Z. J. Psychiatr..

[38] Grant J.E., Odlaug B.L., Kim S.W. (2009). N-acetylcysteine, a glutamate modulator, in the treatment of trichotillomania: a double-blind, placebo-controlled study. Arch. Gen. Psychiatry.

[39] Ooi S.L., Green R., Pak S.C. (2018). N-acetylcysteine for the treatment of psychiatric disorders: a review of current evidence. BioMed Res. Int..

[40] Pesko M.J., Burbige E.M., Sannar E.M., Beresford C., Rogers C., Ariefdjohan M. (2020). The use of N-acetylcysteine supplementation to decrease irritability in four youths with autism spectrum disorders. J Pediatr Pharmacol Ther JPPT.

