# Supplementary material for: Exploring the metabolic signature of intermittent explosive disorder: Preliminary evidence and potential mechanisms for altered bilirubin metabolism
Source: Compr Psychoneuroendocrinol. 2025 Apr 22;22:100294. doi: 10.1016/j.cpnec.2025.100294 (PMC12060501; doi:10.1016/j.cpnec.2025.100294)
Supplement: Multimedia component 1 [file mmc1.docx]

**Supplementary Figure 1: Sensitivity Analyses of Bilirubin and Inflammatory Biomarkers to Unmeasured Confounding in IED vs. GP**

**(a) Indirect Bilirubin**
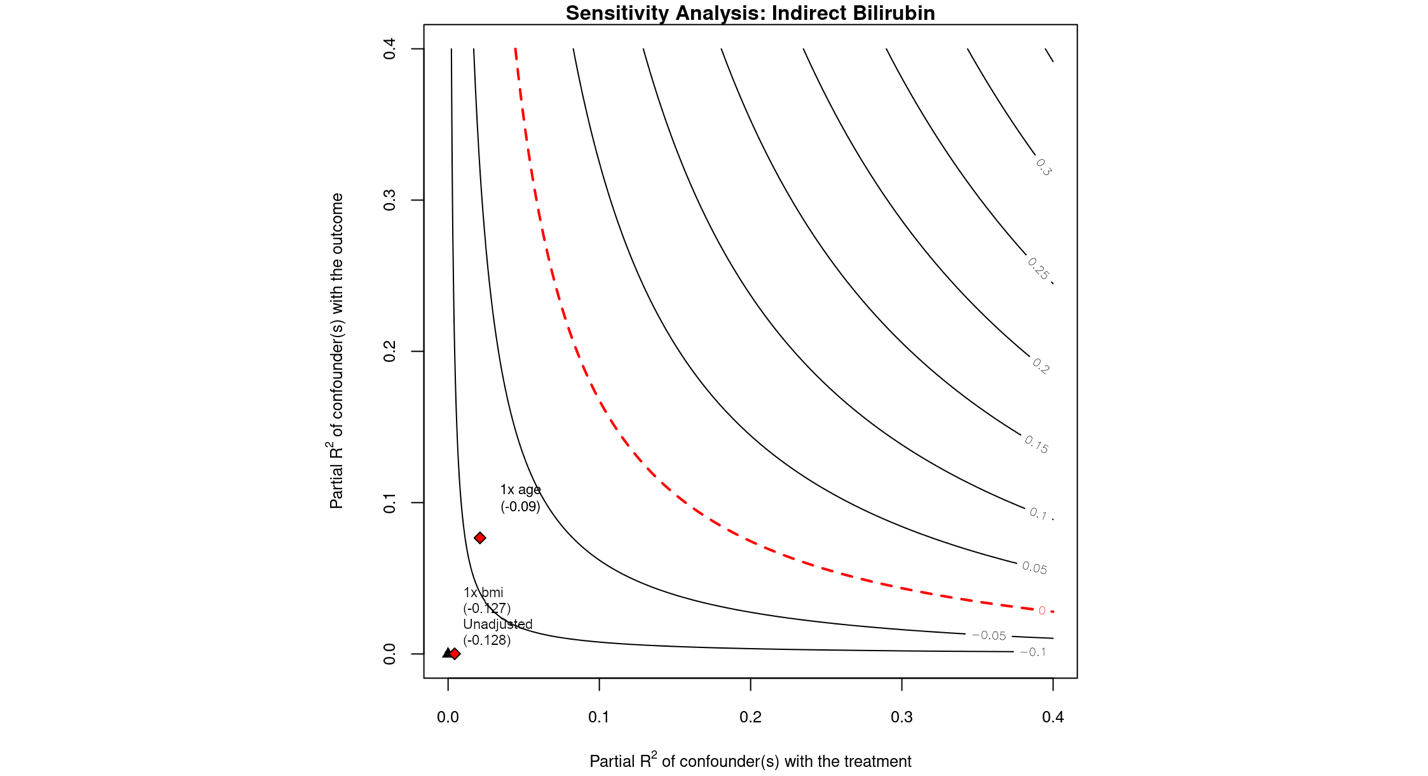


**(b) Total Bilirubin**
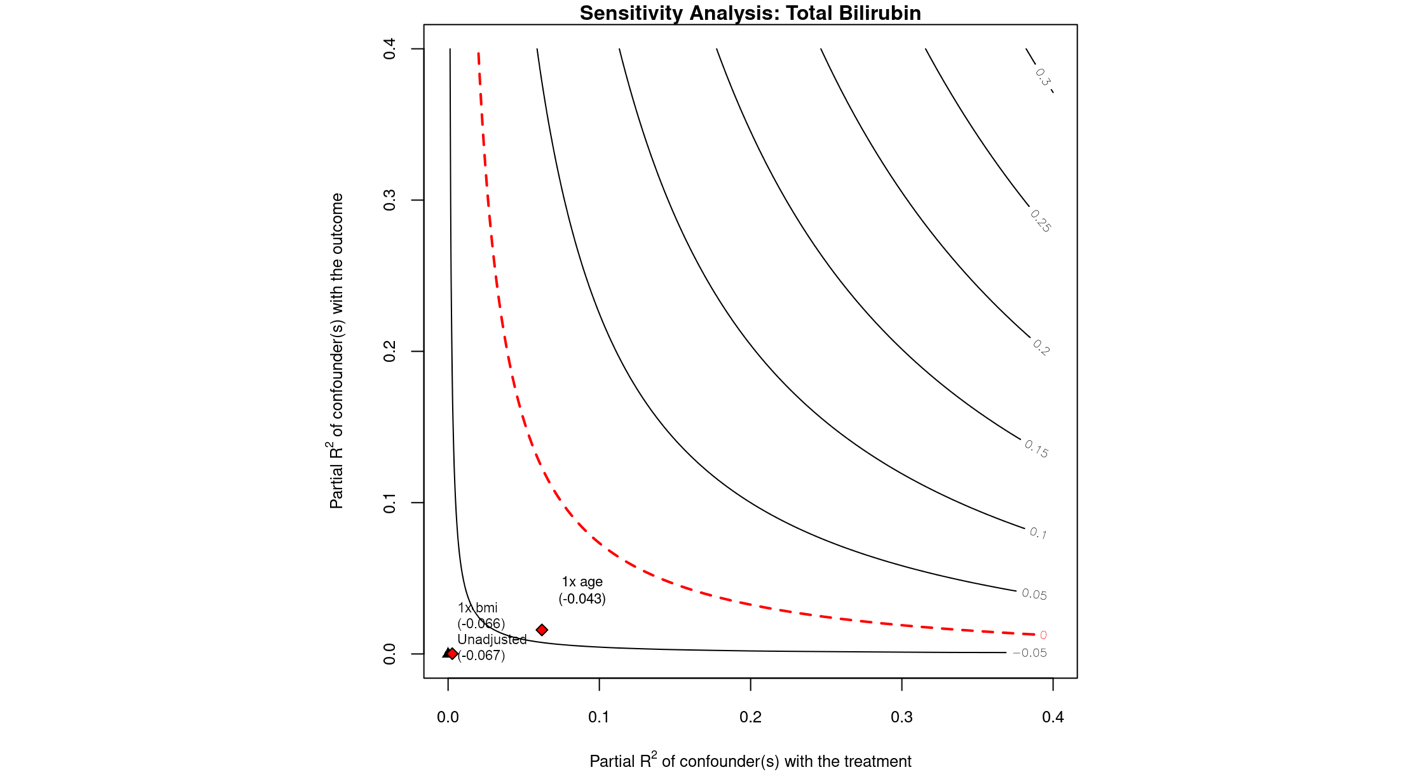


**(c) WBC**
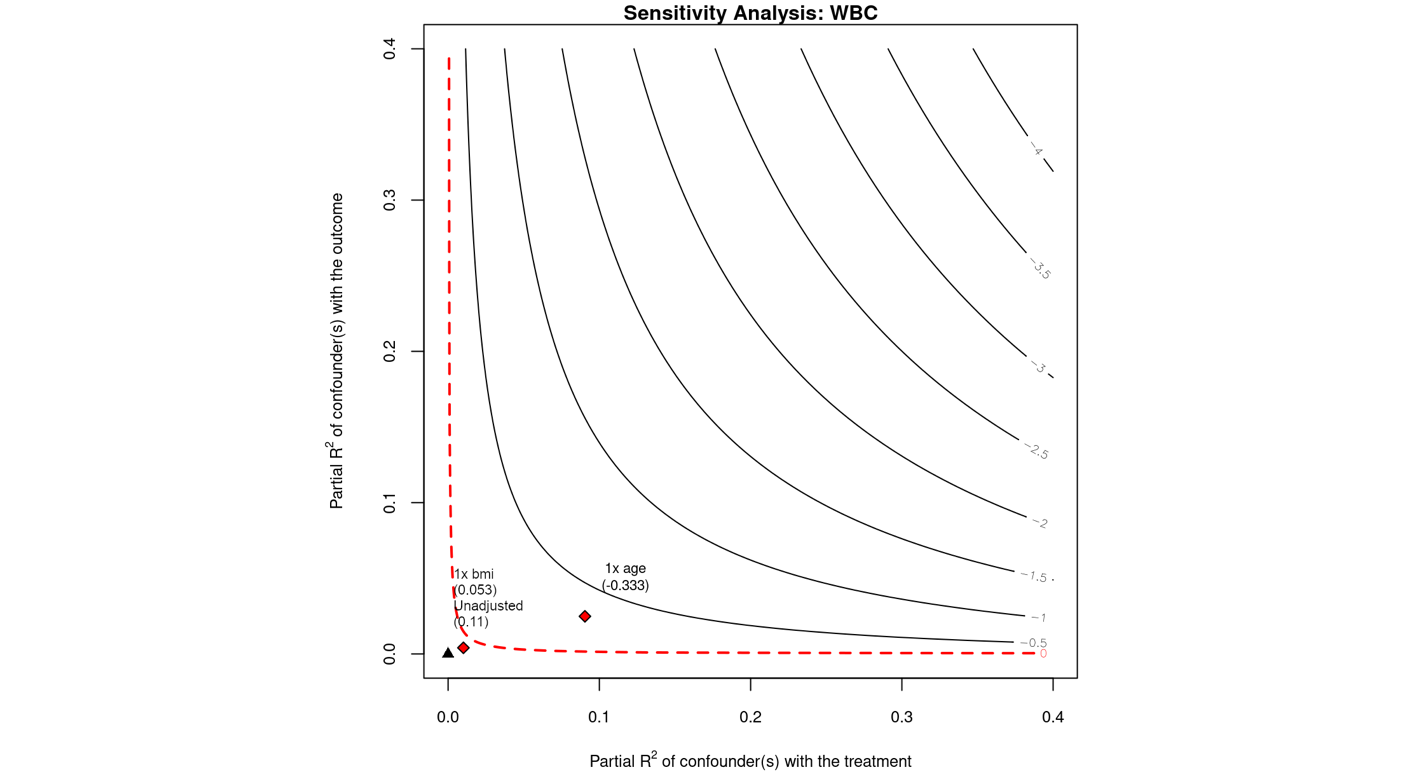


We conducted sensitivity analyses using the sensemakr framework to evaluate the robustness of our findings to potential unmeasured confounding for the three primary biomarkers: indirect bilirubin, total bilirubin, and white blood cell (WBC) count. Supplementary Figure 1 above displays the contour plots illustrating the level of unmeasured confounding required to nullify the observed group differences (IED vs. GP) under varying assumptions. Each plot presents curves representing the estimated effect size under different degrees of unmeasured confounding, quantified in terms of partial R² values with both the treatment and the outcome. The red dashed curve represents the tipping point at which the estimated effect becomes statistically non-significant (p > 0.05), and values to the right of this curve indicate robustness to unmeasured confounding, while those to the left indicate vulnerability.

For indirect bilirubin, the estimated effect of IED was –0.128, with a partial R² of 1.83% for the treatment variable and a robustness value of 12.75%. This indicates that an unobserved confounder would need to account for more than 12.75% of the residual variance in both treatment and outcome to fully explain away the observed association. However, only 0.63% shared variance would be required to render the effect statistically non-significant at the α = 0.05 threshold, highlighting some sensitivity of the p-value to minor unmeasured bias, despite the effect size itself being relatively robust.

Total bilirubin showed a smaller estimated effect of –0.067 and a lower partial R² of 0.81%. As illustrated in the figure above, this effect would require an unobserved confounder accounting for more than 8.63% of residual variance in both the treatment and the outcome to reduce the point estimate to zero. However, the threshold to lose statistical significance was again low, at just 2.05% shared variance. While the magnitude of the group difference in total bilirubin is modest and somewhat more sensitive than that for indirect bilirubin, these results remain consistent with a small but potentially biologically meaningful difference in bilirubin metabolism among individuals with IED.

In contrast, the observed difference in WBC count between groups was negligible and not statistically significant, with a coefficient estimate of 0.11 and a partial R² of only 0.02%. Figure 6 shows that even minimal unmeasured confounding—any variable accounting for as little as 0.01% shared variance—would be sufficient to explain away this association. The robustness value was 1.23%, indicating that the WBC findings lack any meaningful robustness and are most likely attributable to noise or uncontrolled variability rather than systematic group differences.

Taken together, the sensitivity analyses strengthen confidence in the observed bilirubin findings while further supporting the interpretation of null results for WBC. The use of benchmark variables (e.g., age and BMI) on the plots offers visual reference points for understanding how plausible real-world confounders compare in influence to the level of confounding needed to challenge the observed effects.

**Supplementary Figure 2: Residual and Normality Diagnostics for Linear Mixed-Effects Models of Bilirubin and Inflammatory Biomarkers**

**(a) Indirect Bilirubin
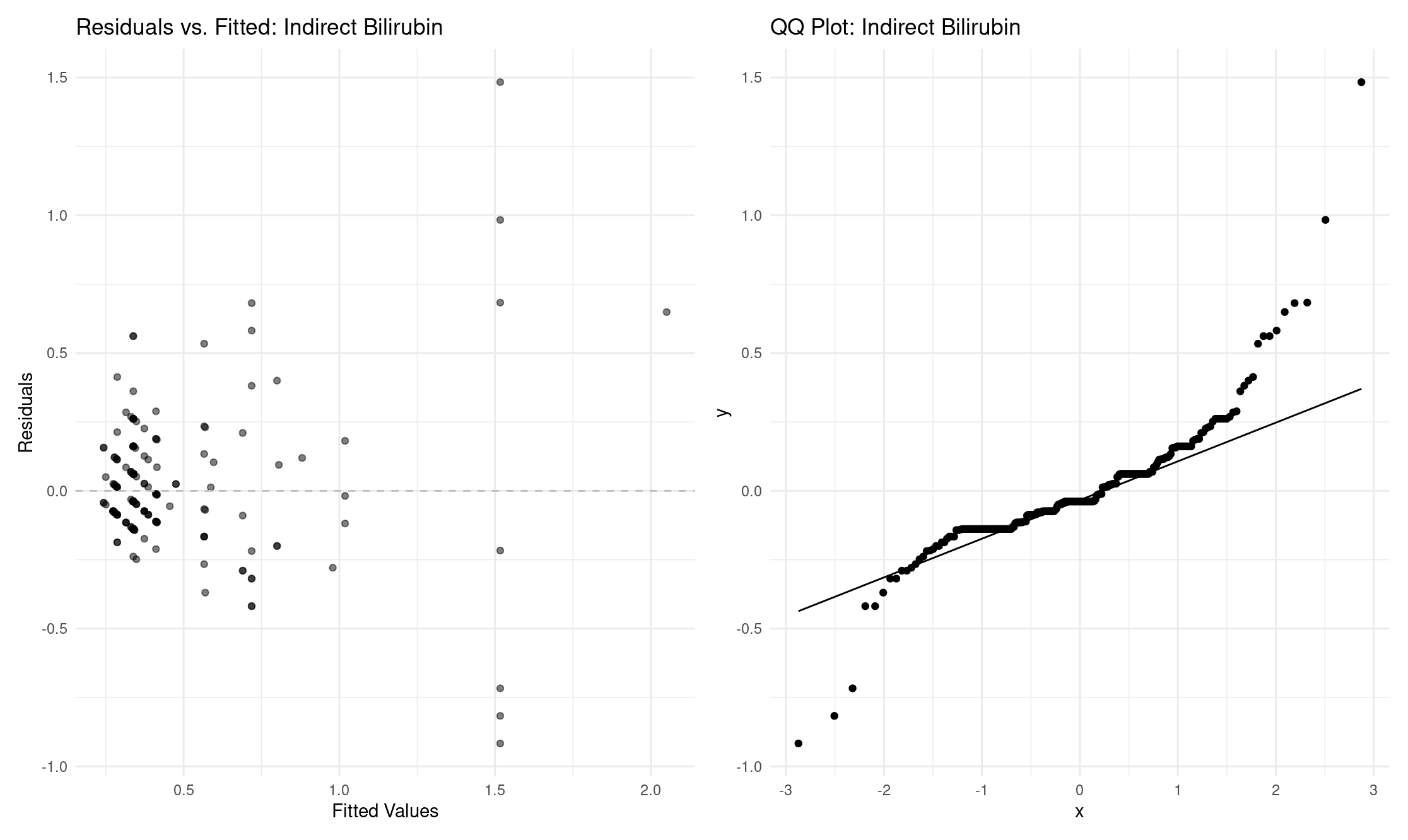
**

**(b) Total Bilirubin**

**
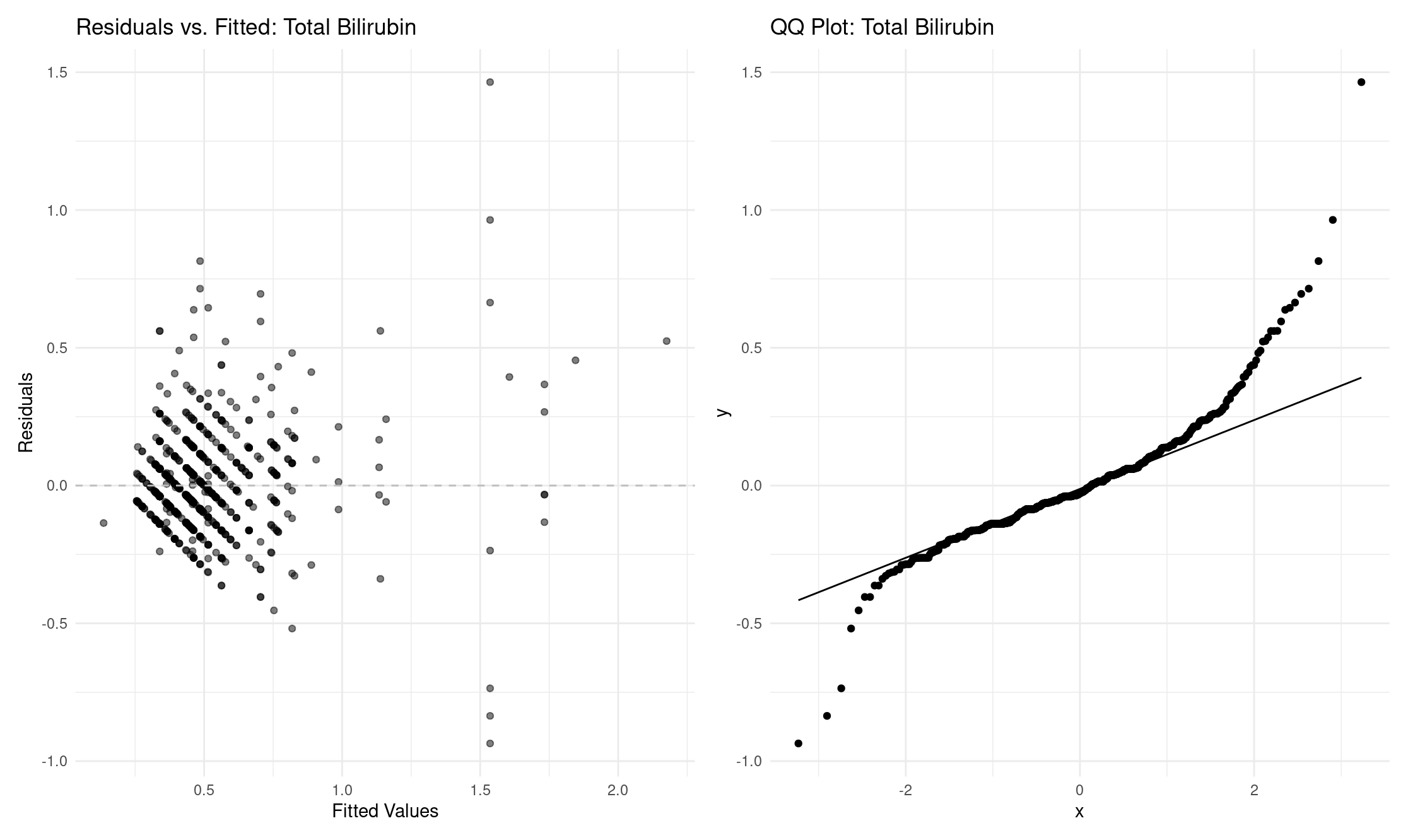
**

**(c) WBC
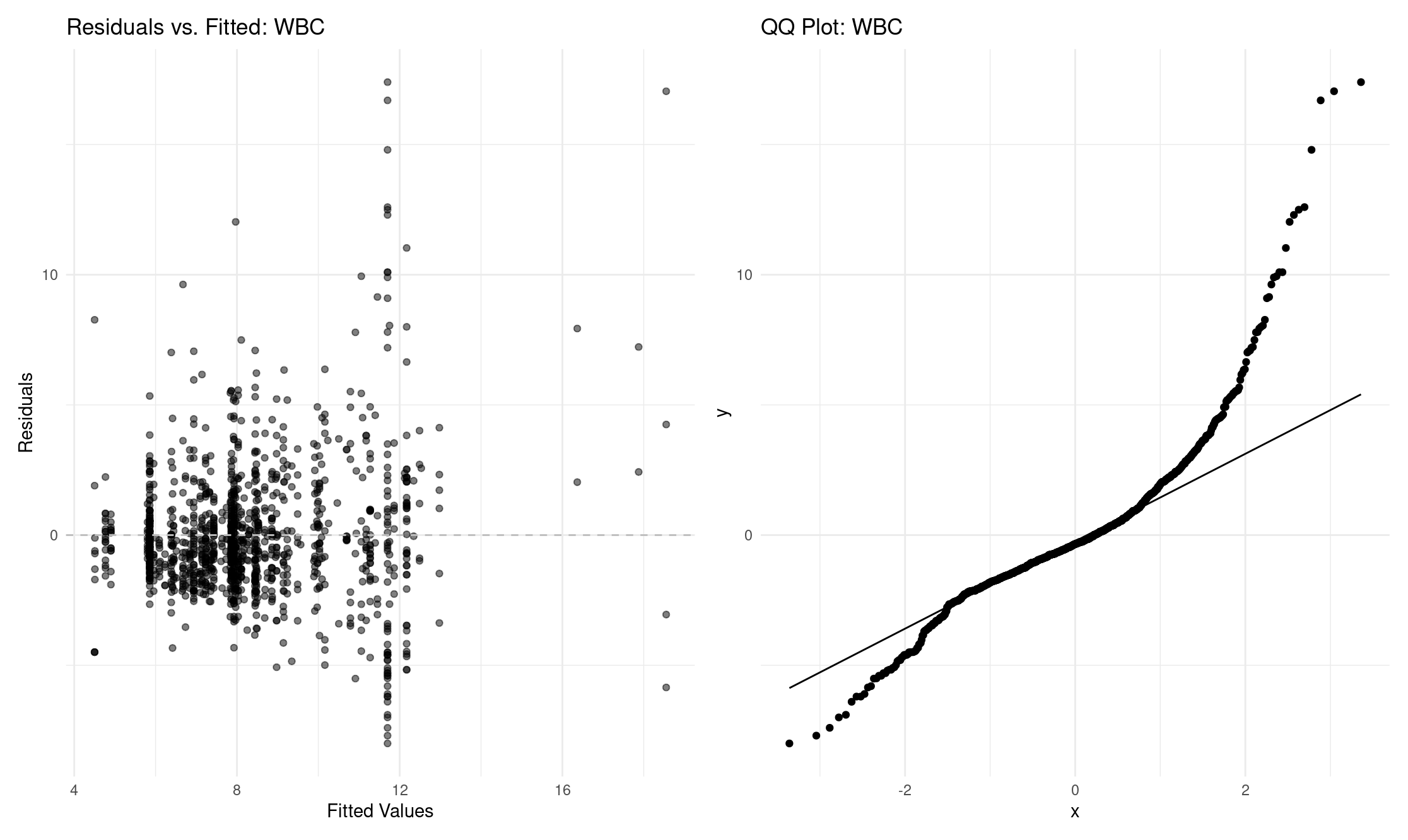
**

The diagnostic plots for the linear mixed-effects models suggest that model assumptions are reasonably met for Indirect and Total Bilirubin, though there are some mild deviations from normality and potential heteroscedasticity. In the residuals vs. fitted plot for Indirect Bilirubin, the spread of residuals appears roughly homoscedastic across fitted values, with no distinct curvature, suggesting linearity and constant variance. However, the Q-Q plot shows mild rightward deviation from the theoretical line at the upper tail, indicating slight positive skew in the residuals.

For Total Bilirubin, the residuals *vs*. fitted plot shows a similarly even scatter, suggesting good model fit and homoscedasticity, while the Q-Q plot again shows a mild right-tail deviation. These results suggest a slight violation of normality in the residuals but do not appear severe enough to invalidate model inference, particularly in larger samples where mixed-effects models are known to be robust to such deviations.

The WBC model, however, presents greater deviations. The residuals *vs.* fitted plot displays considerable heteroscedasticity, with increasing variance at higher fitted values and several extreme residuals. The Q-Q plot also deviates more substantially from the theoretical line, particularly in both tails, indicating non-normality. These findings suggest that the WBC model may be influenced by outliers or a non-Gaussian error structure. WBC was not subjected to additional cleaning (e.g., outlier removal) because the signal was already sensitive to confounding as described above in Supplementary Figure 1(c).
